# KIT is dispensable for physiological organ vascularisation in the embryo

**DOI:** 10.1007/s10456-022-09837-6

**Published:** 2022-04-13

**Authors:** Carlotta Tacconi, Alice Plein, Chiara Colletto, Emanuela Villa, Laura Denti, Cristiana Barone, Yousef Javanmardi, Emad Moeendarbary, Emanuele Azzoni, Alessandro Fantin, Christiana Ruhrberg

**Affiliations:** 1 Department of Biosciences, University of Milan, Via G. Celoria 26, 20133 Milan, Italy; 2 UCL Institute of Ophthalmology, University College London, 11-43 Bath Street, London, EC1V 9EL UK; 3 Present Address: Department of Experimental Oncology, IEO, European Institute of Oncology IRCCS, Milan, Italy; 4 School of Medicine and Surgery, University of Milano-Bicocca, Monza, Italy; 5 UCL Department of Mechanical Engineering, University College London, London, UK

**Keywords:** KIT, Angiogenesis, Development

## Abstract

Blood vessels form vast networks in all vertebrate organs to sustain tissue growth, repair and homeostatic metabolism, but they also contribute to a range of diseases with neovascularisation. It is, therefore, important to define the molecular mechanisms that underpin blood vessel growth. The receptor tyrosine kinase KIT is required for the normal expansion of hematopoietic progenitors that arise during embryogenesis from hemogenic endothelium in the yolk sac and dorsal aorta. Additionally, KIT has been reported to be expressed in endothelial cells during embryonic brain vascularisation and has been implicated in pathological angiogenesis. However, it is neither known whether KIT expression is widespread in normal organ endothelium nor whether it promotes blood vessel growth in developing organs. Here, we have used single-cell analyses to show that KIT is expressed in endothelial cell subsets of several organs, both in the adult and in the developing embryo. Knockout mouse analyses revealed that KIT is dispensable for vascularisation of growing organs in the midgestation embryo, including the lung, liver and brain. By contrast, vascular changes emerged during late-stage embryogenesis in these organs from KIT-deficient embryos, concurrent with severe erythrocyte deficiency and growth retardation. These findings suggest that KIT is not required for developmental tissue vascularisation in physiological conditions, but that KIT deficiency causes foetal anaemia at late gestation and thereby pathological vascular remodelling.

**Supplementary Information:**

The online version contains supplementary material available at 10.1007/s10456-022-09837-6.

## Introduction

Endothelial cells (ECs) form the inner lining of blood vessels, which enable organ health by supplying nutrients and oxygen whilst removing waste materials. In the embryo, ECs first differentiate from mesenchymal precursors termed angioblasts, both in the extra-embryonic yolk sac and in the lateral plate mesoderm of the embryo proper [1]. These early ECs condense into the yolk sac vasculature and dorsal aortae, respectively, in a process termed vasculogenesis. Thereafter, ECs proliferate within blood vessels and either become migratory to expand the vasculature by sprouting angiogenesis or form tissue pillars for intussusceptive angiogenesis [1].

In close spatiotemporal proximity to blood vessel formation, several consecutive waves of hematopoietic progenitors arise and contribute blood and immune cells to the growing vertebrate embryo [2]. Whereas the first of these progenitors differentiate alongside ECs in yolk sac blood islands, subsequent waves of hematopoietic progenitors arise from so-called hemogenic endothelium that undergoes an endothelial-to-hematopoietic transition (endoHT) [2]. Work in the mouse as a mammalian model organism revealed that the progeny of yolk sac endoHT, termed erythromyeloid progenitors (EMPs), gives rise to the early tissue macrophages and also to erythrocytes, but later born EMPs leave the yolk sac to colonise the liver via the blood stream [2]. These liver-resident EMPs give rise to monocytes, megakaryocytes and erythroid progenitors that gradually replace primitive erythrocytes to then sustain erythropoiesis until birth. More recently, we observed that cells with EMP characteristics also contribute ECs to organ vasculature and that *Hoxa* gene cluster ablation in this cell lineage reduced brain angiogenesis [3], although different genetic tools to lineage trace EMP-derived cells have yielded conflicting results as to the prevalence of EMP-derived ECs [4].

Cell surface expression of the receptor tyrosine kinase KIT is a key marker for EMPs [5–7] and hematopoietic stem cells (HSCs) [6, 8]. Moreover, KIT cell surface expression has been proposed as a distinguishing feature of blood forming (hemogenic) ECs in the embryonic yolk sac and dorsal aorta, which give rise to hematopoietic progenitors during endoHT [9]. We have recently shown that KIT surface expression in the yolk sac is restricted to cells that are already budding from the yolk sac endothelium and which have internalised the EC junctional protein CDH5, suggesting that KIT marks cells which have abandoned their EC identity to acquire EMP features [10]. KIT expression has recently also been reported in embryonic brain endothelium [4], corresponding to the time when the brain is first vascularised by sprouting angiogenesis [11, 12]. Moreover, KIT has been implicated in EC migration, proliferation and tube formation through in vitro studies [13–15], and decreased KIT expression reduces angiogenesis in mouse models of ocular pathology [15] and tumours [16]. However, it has not previously been examined whether KIT is expressed in the endothelium of other developing organs other than the brain nor whether KIT is required for organ vascularisation.

Here, we have combined single-cell expression studies with confocal imaging and the analysis of knock out mice to investigate endothelial *Kit* expression in several adult and embryonic organs and examined organ vascularisation in mouse embryos lacking KIT.

## Results

### KIT is expressed in adult liver and lung ECs but not brain ECs

**Fig. 1:**
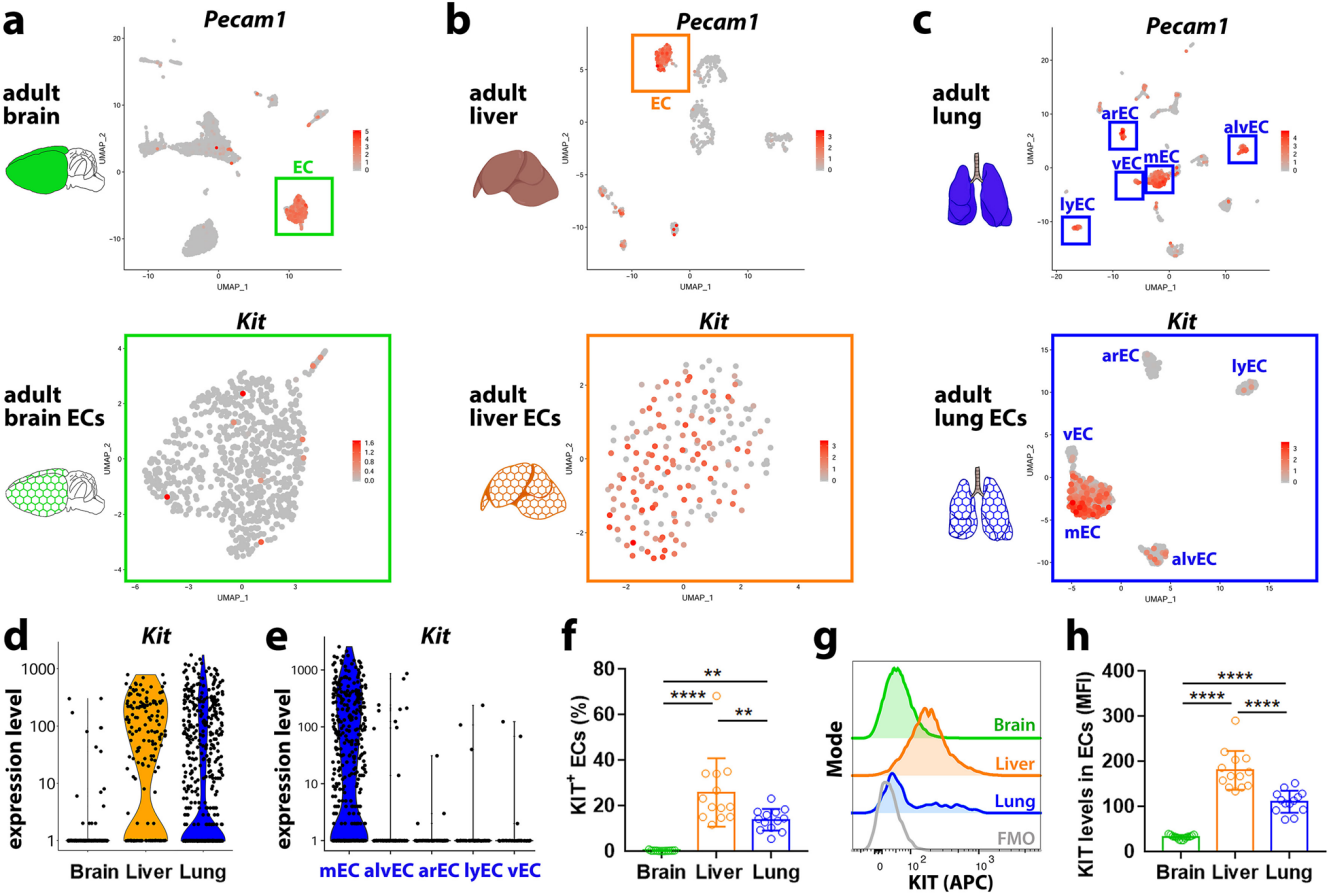
*Kit* mRNA and KIT protein expression in ECs of the adult brain, liver and lung. **a**–**e** scRNA-seq analysis of adult mouse brain (**a**), liver (**b**) and lung (**c**), including schematic representations of each organ. UMAP plots show *Pecam1* transcript levels in the total cell population for each organ (**a-c**, top panels), whereby *Pecam1* expression was used to select the EC clusters for *Kit* transcript visualisation (**a-c**, bottom panels). Violin plots (**d,e**) compare *Kit* transcript levels in brain, liver and lung ECs (**d**) and in the different lung EC subsets (**e**). mEC, microvascular ECs; alvEC, alveolar ECs; arEC, arterial ECs; lyEC, lymphatic ECs; vEC, venous ECs. **f**–**h** Flow cytometry analysis for KIT protein on the surface of single, living ECs from adult mouse organs, *n* = 13 for each organ from a total of 3 independent experiments. **f** Proportion of KIT^+^ ECs in all ECs of each organ. **g**, **h** Representative plot profile (**g**) and quantification (**h**) of KIT protein surface levels in ECs; FMO, fluorescence minus one. The percentage of KIT^+^ ECs and KIT’s mean fluorescent intensity (MFI) in ECs are shown as mean ± SD; each data point represents the value from one embryo; ** *p* < 0.01, **** *p* < 0.0001 (one-way ANOVA followed by Tukey's multiple comparisons test)

*Kit* has previously been shown to be expressed in adult hepatic sinusoidal ECs [17, 18] and in subsets of adult lung ECs [19, 20]. Taking advantage of the Tabula Muris single-cell RNA sequencing (scRNA-seq) database [21], we reduced dimensionality and applied Uniform Manifold Approximation and Projection (UMAP) to the datasets from adult brain, liver and lung to select the clusters containing *Pecam1*^+^ ECs and compare *Kit* transcript levels (Fig. 1a–c). We observed that adult brain and liver ECs clustered each into one main population, with arterial and venous ECs localising to opposite sides of the same cluster (Fig. S1a, b). In the adult liver, *Kit*^+^ ECs were distributed throughout the EC cluster, whereas *Kit*^+^ ECs were extremely rare in the adult brain (Fig. 1a, b, d). In contrast to the brain and liver, we identified 5 discrete clusters of *Pecam1*^+^ ECs in the adult lung that comprised ECs with an arterial identity (arEC, *Bmx*^+^), ECs with a venous identity (vEC, *Nr2f2*^+^), lymphatic ECs (lyEC, *Prox1*^+^), alveolar ECs (alvEC, *Car4*^+^) and microvascular ECs (mEC, *Sema3c*^+^ *Aplnr*^+^) (Fig. 1c, e; Fig. S1c), similar to previous reports using different datasets [19, 22–24]. *Kit* transcripts were highly enriched in microvascular ECs and only rarely present in the other lung EC subtypes (Fig. 1c, e). Agreeing with the transcriptomic data showing abundant *Kit*^+^ cells in adult liver and lung but not brain (Fig. 1d), flow cytometry demonstrated that nearly all brain ECs, defined as PECAM1 (CD31)-positive and CD45, CD41, CD11b-triple negative cells (Fig. S1d), lacked KIT protein on their surface (Fig. 1f; Fig. S1d) or expressed KIT at very low levels (Fig. 1g, h; Fig. S1d). By contrast, ~ 30% of lung ECs and ~ 15% of liver ECs were KIT^+^ (Fig. 1f; Fig. S1d). Also agreeing with the transcriptomic analysis, which had identified *Kit* expression in microvascular but no other lung EC subpopulations (Fig. 1c, d), KIT surface levels distinguished a positive from a negative EC cluster in the lung (Fig. 1g). Taken together, the *Kit* expression profile raises the possibility that KIT might have organ-specific roles in ECs.

### *Kit* expression in embryonic organ ECs

**Fig. 2:**
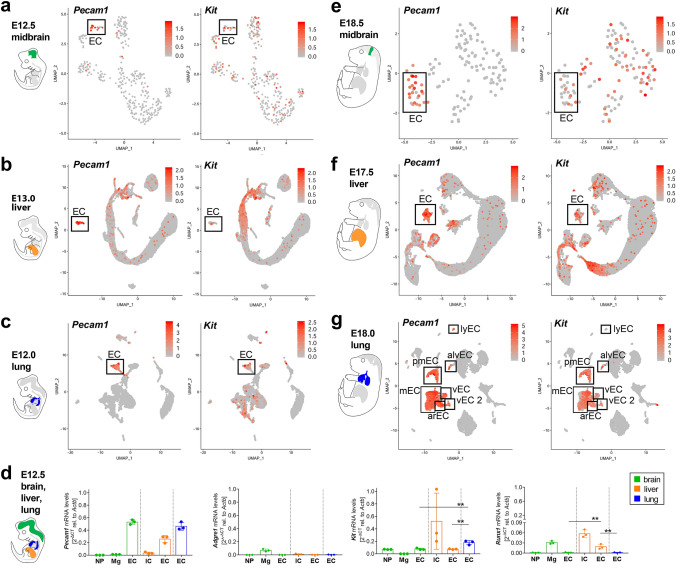
*Kit* expression in ECs of the brain, liver and lung in mid and late gestation embryos. **a**–**c**, **e**–**g** scRNA-seq analysis of mouse E12.5 (**a**) and E18.5 (**e**) midbrain, E13.0 (**b**) and E17.5 (**f**) liver, E12.0 (**c**) and E18.0 (**g**) lung, including a schematic representation to illustrate the organ analysed. UMAP plots visualise *Pecam1* (left panels) and *Kit* transcript levels (right panels) in each cell cluster; the boxes indicate the EC clusters. **d** qRT-PCR analysis of *Pecam1, Adgre1* (F4/80), *Kit* and *Runx1* expression in the indicated cell populations isolated by FACS from E12.5 mouse brain, liver and lung (*n* = 3 each). All bar graph data are shown as mean ± SD; each data point represents the value from three pooled embryos from the same litter (brain and liver) or from all the embryos of one litter (lung). IC, immune cells; Mg, microglia; NP, neural parenchyme

*Kit* transcripts and KIT protein were recently described in embryonic brain endothelium [4]. To corroborate these findings and place them into the context of vascular development in the midgestation mouse embryo, we analysed publicly available scRNA-seq datasets from the E12.5 midbrain (GSE76381) [25], E13.0 liver (CRA002445) [26] and E12.0 lung (GSE165063) [27]. For all datasets, we performed graph-based clustering and identified the EC clusters as *Pecam1*^+^, *Cdh5*^+^ and *Cldn5*^+^ (Fig. 2a–c; Fig. S2). Consistent with active sprouting angiogenesis via endothelial tip cells in the E12.5 brain [11, 12, 28], the EC cluster included cells with transcripts for the endothelial tip cell markers *Dll4* and *Apln* as well as the proliferation markers *Pcna* and *Mki67* (Fig. S2d). Some brain ECs contained *Kit* transcripts but did not contain detectable *Runx1* transcript levels (Fig. 2a; Fig. S2d, f). Our recent scRNA-seq analysis had also identified rare foetal liver ECs with low levels of *Kit* or *Runx1* [10], and we confirmed this observation here at E13.0 (Fig. 2b; Fig. S2j). The analysis of scRNA-seq data from E12.0 lung showed that some lung ECs expressed *Kit*, but we could not detect *Runx1* transcripts in lung ECs (Fig. 2c; Fig. S2n). In agreement with the scRNA-seq data, quantitative RT-PCR (qRT-PCR) of FACS-isolated brain, liver and lung cell populations identified *Kit* transcripts in brain and liver ECs, albeit in lower amounts compared to lung ECs (Fig. 2d; Fig. S3). *Kit* is, therefore, expressed in ECs of all three embryonic organs at a time when they are vascularised. qRT-PCR identified *Runx1* transcripts at negligible levels in brain and lung ECs and at relatively higher levels in brain microglia and liver immune cells (Fig. 2d). Liver ECs appeared to express low levels of *Runx1*, but higher compared to brain or lung ECs (Fig. 2d). Lack of *Runx1* expression in brain or lung ECs argued against KIT expression being an indicator of a hemogenic state in these organs, because *Runx1* is normally co-expressed with *Kit* in hemogenic ECs in the yolk sac and dorsal aorta [2, 29, 30]. Rare *Runx1* transcripts in foetal liver ECs were unexpected, because the foetal liver harbours, rather than produces, hematopoietic progenitors [2, 5, 7]. To understand whether KIT was expressed in ECs at later stages of vascular development, we analysed publicly available scRNA-seq datasets for the E18.5 midbrain (GSE76381) [25], E17.5 liver (CRA002445) [26] and E18.0 lung (GSE160876). Our analysis identified one EC cluster in the midbrain and liver datasets, with ECs containing *Kit* (Fig. 2e, f; Fig. S4a, b). By contrast, we identified several different subpopulations of ECs in the lung, with *Kit* enriched in both proliferating and non-proliferating lung microvascular ECs, but not the other EC subtypes (Fig. 2g; Fig. S4c), similar to the adult lung (Fig. 1). Together, these findings demonstrate *Kit* expression in embryonic organ ECs during the period of blood vessel expansion.

### KIT protein is present in a subset of embryonic brain, liver and lung ECs

**Fig. 3:**
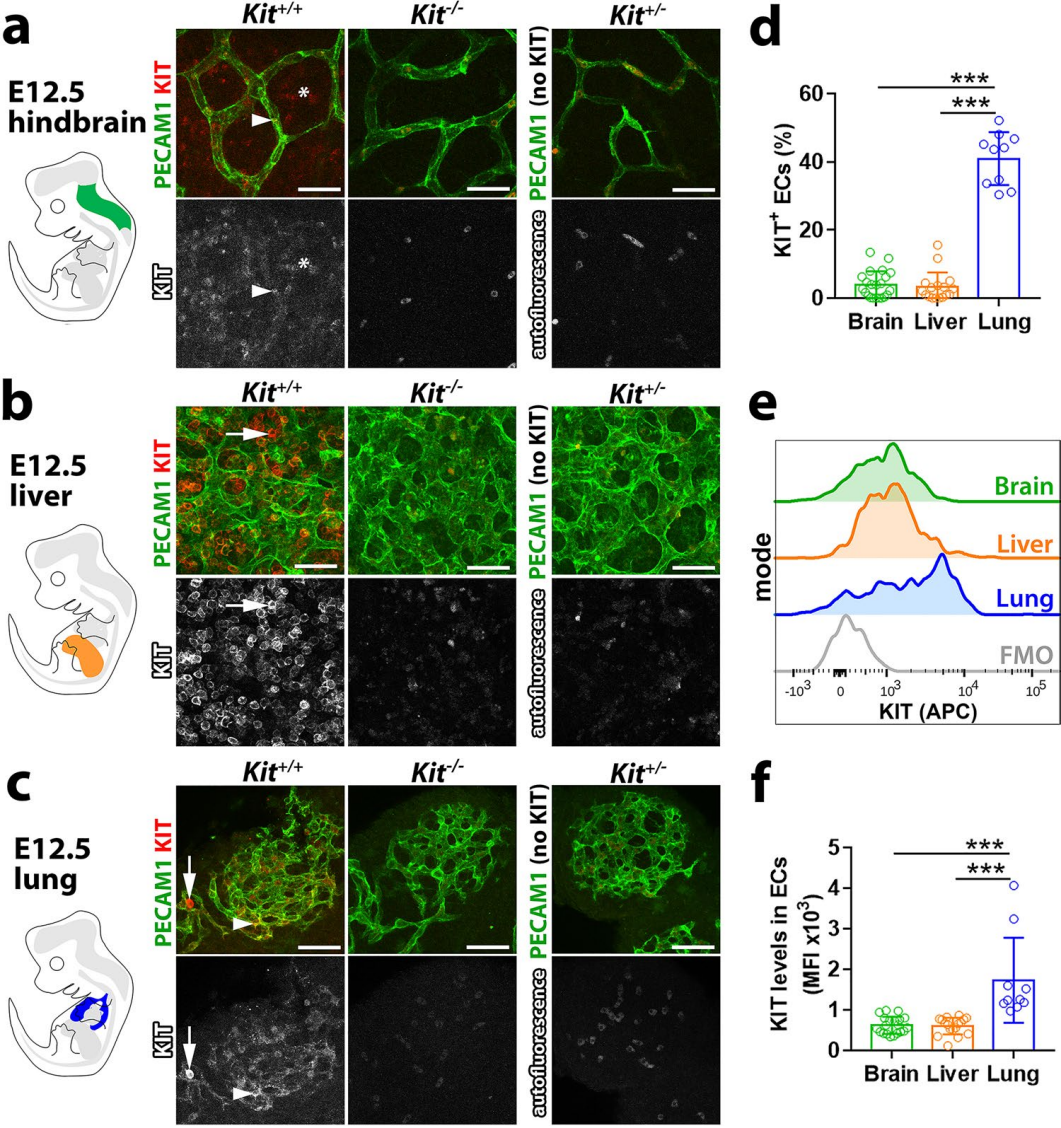
KIT protein expression in embryonic brain, liver and lung. **a**–**c** E12.5 mouse hindbrain (**a**), liver (**b**) and lung (**c**) from *Kit*^+*/*+^ (*n* = 4), *Kit*^*−/−*^ (*n* = 4) and *Kit*^±^ (*n* = 3) embryos from 2 litters were immunostained with the indicated markers and imaged by confocal microscopy. Single-channel images for KIT are also shown in grey scale. Arrowheads indicate KIT^+^ ECs, arrows indicate KIT^+^ hematopoietic cells and the asterisk indicates KIT^+^ neuroepithelium. Note that the signal in the KIT channel of *Kit*^*−/−*^ samples corresponds to autofluorescence, which is also observed in unstained samples (*Kit*^±^). Scale bar: 50 µm. **d**–**f** Flow cytometry analysis for KIT protein on the surface of single-living ECs from E12.5 mouse organs isolated from embryos across 6 independent experiments, brain (midbrain and hindbrain) *n* = 22, liver *n* = 17 and lung *n* = 10. **d** Quantification of the proportion of KIT^+^ ECs in all ECs of each organ. **e**, **f** Representative plot profile (**e**) and quantification (**f**) of KIT protein surface levels in ECs; FMO, fluorescence minus one. The percentage of KIT^+^ ECs and KIT’s mean fluorescent intensity (MFI) in ECs are shown as mean ± SD; each data point represents the value from one embryo; ***p < 0.001 (one-way ANOVA followed by Tukey’s multiple comparisons test)

The presence of *Kit* transcripts raised the possibility that KIT protein might also be present in ECs during organ vascularisation. Wholemount immunostaining showed that KIT protein was present at low levels in E12.5 brain ECs (Fig. 3a), consistent with our transcriptomic analysis (Fig. 2). By contrast, wholemount immunostaining did not detect obvious KIT protein in liver ECs at this stage, possibly masked by the great abundance of round, hematopoietic-like cells with high KIT levels (Fig. 3b, arrow). The lungs also contained scattered KIT^+^ hematopoietic-like cells (Fig. 3c, arrow), but these were much rarer than in the liver (compare Fig. 3b with 3c). Vice versa, KIT staining was more prominent in E12.5 lung than brain or liver ECs (Fig. 3c; arrowheads). For flow cytometry at E12.5, we defined ECs as PECAM1^+^ and CD45/CD41/CD11b-triple negative or CD45/CD11b-double negative cells. First, we found that fewer than 5% of brain ECs had KIT surface protein (Fig. 3d; Fig. S5a, b) and KIT surface levels were low in these cells (Fig. 3e, f; Fig. S5a, b). Accordingly, despite *Kit* transcripts being present in angiogenic brain ECs (Fig. 2), most cells lacked KIT protein. Second, the number of KIT^+^ ECs in the E12.5 liver and KIT expression levels were also low (Fig. 3d–f; Fig. S5a, b). Third, the number of KIT^+^ ECs was higher in E12.5 lung than in brain and liver, with 40% of lung ECs KIT^+^ at this stage (Fig. 3d; Fig. S5a, b). KIT surface levels were also higher in E12.5 lung compared to brain or liver ECs (Fig. 3e, f; Fig. S5a, b). Flow cytometry analysis of brain, liver and lung ECs during late-stage embryogenesis yielded similar results. Thus, ~ 50% of E16.5 and ~ 23% of E18.5 lung ECs were KIT^+^ (Fig. S5c), but KIT^+^ ECs made only a small contribution to the EC populations in E16.5 and 18.5 brain and liver (< 10%; Fig. S5c), and KIT surface levels were also higher in E16.5 and E18.5 lung compared to brain or liver ECs (Fig. S5a, b). Together, these findings suggest that KIT might act in a subset of ECs to promote embryonic organ vascularisation, particularly in the lung.

### KIT is not required for physiological embryonic organ vascularisation

**Fig. 4:**
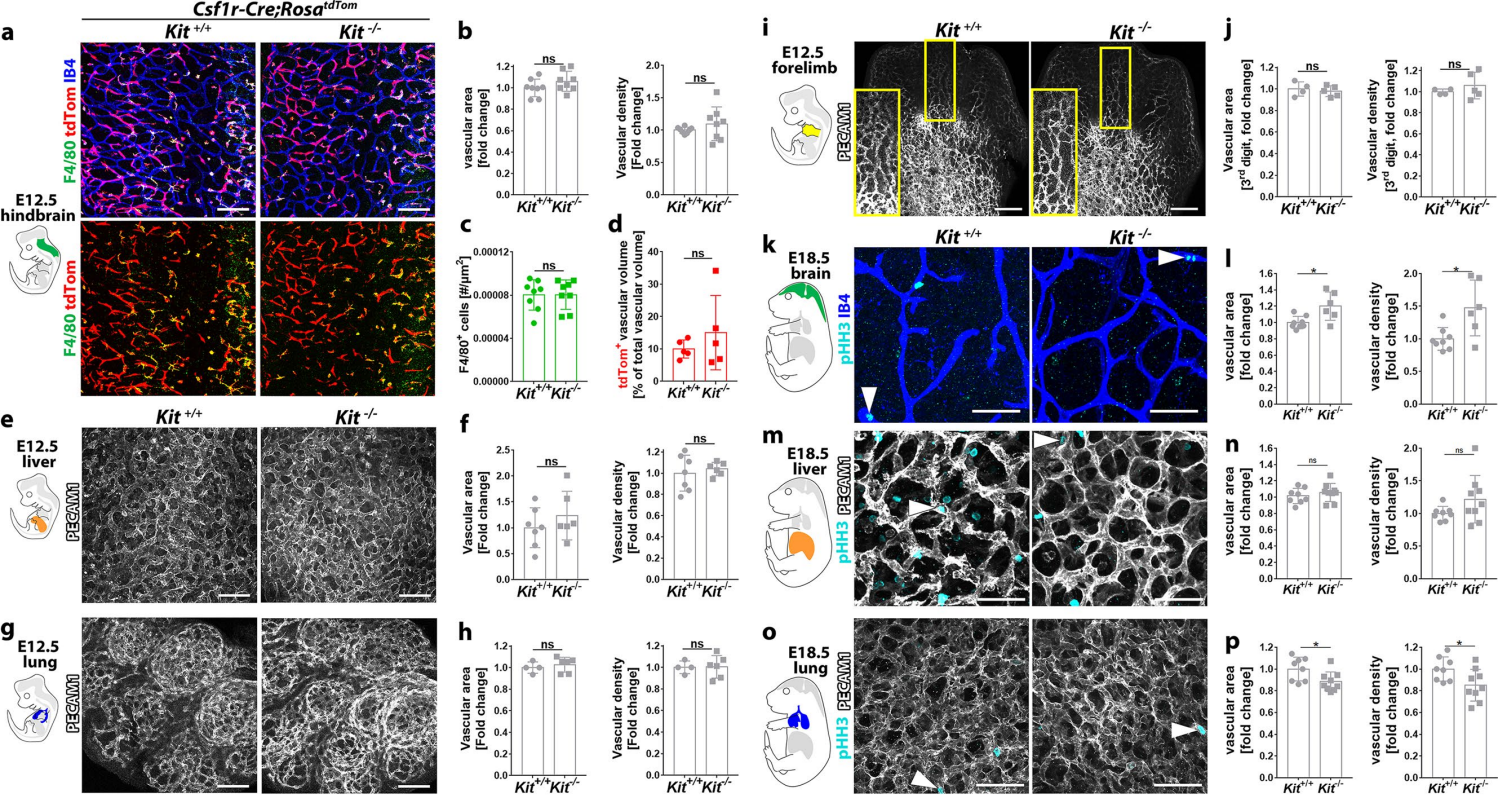
Impact of KIT loss on organ vascular density in embryogenesis. Confocal z scans and quantification of vascular parameters in the indicated organs from E12.5 (**a**–**j**) and E18.5 (**k**–**p**) *Kit*^+*/*+^ versus *Kit*^*−/−*^ littermates after staining with the indicated markers; scale bars: 200 µm (**a**–**g**); 50 µm (**k**–**o**). In **a**, hindbrains also carrying the *Csf1r-iCre;Rosa*^*tdTom*^ reporter are shown with the IB4, F4/80 and tdTomato (tdTom) channels in the top panels and without IB4 in the bottom panels. In **b**, **c**, we quantified vascular area and density (**b**) as well as microglia number (**c**) in *n* = 8 wild type and mutant hindbrains each from 3 litters. In **d**, we analysed the percentage of lineage-traced endothelium in *n* = 5 wild type and mutants each from 3 litters. **f**, **h**, **j** Vascular area and density quantification in *n* = 7 wild type and 6 mutant livers from 3 litters (**f**), *n* = 4 wild type and 6 mutant lungs from 2 litters (**h**) and *n* = 4 wild type and 5 mutant forelimbs from 2 litters (**j**). The area highlighted in (**i**) with a yellow box corresponds to the middle digit and is shown at higher magnification in the inset; this area also corresponds to the area used for quantification in (**j**). **l**, **n**, **p** Vascular area and density quantification in *n* = 8 for all wild-type organs, *n* = 9 mutant livers and lungs and *n* = 6 mutant brains from 3 litters. The arrowheads indicate examples of phosphorylated histone H3 (pHH3)-positive ECs. Data are shown as mean ± SD; each data point represents the value from one embryo; ns, non-significant (unpaired Student’s *t* test)

Up to E12.5, oxygen transport is sustained predominantly by primitive erythrocytes [31], which arise in the yolk sac from KIT-independent progenitors [10]. Accordingly, we examined the E12.5 brain, liver and lung to determine whether KIT expression by ECs was required for organ vascularisation, independently of KIT roles in erythropoiesis. First, we analysed the hindbrain as an established angiogenesis model [11]. Fluorescent wholemount staining for the brain vascular endothelial marker isolectin B4 (IB4) revealed similar vascular area and complexity in *Kit*^+*/*+^ and *Kit*^*−/−*^ hindbrains (Fig. 4a, b). As microglia contribute to brain vascularisation [28], we also examined the number of F4/80^+^ microglia, but found it to be unchanged in *Kit*^*−/−*^ compared to *Kit*^+*/*+^ hindbrains (Fig. 4a, c), as recently shown by CSF1R immunostaining [10]. We also compared the contribution of *Csf1r-iCre* lineage ECs to *Kit*^+*/*+^ and *Kit*^*−/−*^ hindbrains, because KIT^+^ *Csf1r*^+^ cells contribute to brain angiogenesis [3]. However, the proportion of tdTomato^+^ ECs in *Csf1r-iCre;Rosa*^*tdTom*^ hindbrain vasculature was also similar in *Kit*^+*/*+^ and *Kit*^*−/−*^ hindbrains (Fig. 4a, d). Normal numbers of microglia and *Csf1r-iCre* lineage EC cells agree with the lack of angiogenesis defects in the KIT-null hindbrain. We next examined angiogenesis in the embryonic lung and liver by PECAM1 immunostaining. This analysis showed similar vascular area and density in both organs of *Kit*-null mutants compared to control littermates (Fig. 4e–h). Finally, we examined the forelimb, which has previously been used to study neuronal and vascular co-patterning [32]. However, PECAM1 immunostaining also showed similar vascular area and density in the E12.5 forelimb (Fig. 4i, j). These findings suggest that KIT loss does not impair organ vascularisation in the embryo at a time when KIT is expressed by a subset of ECs and KIT-independent erythropoiesis still sustains embryo growth [10].

### Impaired foetal liver erythropoiesis concurs with abnormal organ vascular patterning

After E12.5, oxygen transport is increasingly sustained by KIT-dependent foetal liver erythrocytes [10], which gradually replace primitive erythrocytes [31, 33]. Accordingly, mice homozygous for the *Kit*^*IRES:CreERT2*^ knock-in allele [34], in which the *Cre* recombinase gene is inserted into the endogenous *Kit* locus to generate a true *Kit*-null allele [35], show defective liver erythropoiesis [10]. In agreement, we found that E16.5 and E18.5 KIT-null embryos appeared pale, with smaller body and liver size (Fig. S6a). Similar phenotypes (Fig. S6b) were present in homozygous embryos for an independently generated KIT-null allele termed *Kit*^*MerCreMer*^ [36]. These defects included a severe reduction in TER119^+^ erythroid cells in all organs examined (Fig. S6c-e), as well as a smaller body size and reduced cellularity in the liver and lung, reflecting growth retardation (Fig. S6d, e), consistent with erythrocyte deficiency impairing tissue oxygenation. Notably, the head size of all E16.5 and E18.5 KIT-null embryos examined was relatively less affected than the rest of the body, and their brain cellularity was not significantly reduced compared to that of control littermates (Fig. S6a–c). This growth pattern is reminiscent of that observed in human foetuses with intrauterine growth restriction due to an impaired vascular supply. For subsequent analyses of organ vascular patterning in the anaemic foetus, we, pooled results from both types of KIT-null mutants at late gestational stages and found that their vascular morphogenesis was slightly affected. We found that mutant brains showed increased vascular area and density (Fig. 4k,l) but similar expression of the hypoxia-regulated *Vegfa* and *Hif1a* genes and similar EC proliferation when compared to control brains (Fig. S6f, g). Liver blood vessels in mutants at this late gestational stage showed a slight but insignificant increase in vessel density and EC proliferation (Fig. 4m, n; Fig. S6f) and had a reduced diameter (*Kit*^+*/*+^ 31.28 ± 5.21, *n* = 5; *Kit*^*−/−*^ 21.93 ± 1.1, *n* = 8; *p* < 0.001). Further, the vascular area and density were reduced in mutant lungs, and so was EC proliferation, albeit not significantly (Fig. 4o, p; Fig. S6f). Notably, the presence of vascular defects did not mirror the proportion of KIT^+^ ECs in these organs (compare Fig. S5 with Fig. 4). We conclude that impaired foetal liver erythropoiesis due to KIT loss concurs with abnormal organ vascular patterning at late gestational stages, whereby the hematopoietic KIT requirement likely affects vascular morphogenesis in an indirect manner.

## Discussion

KIT has previously been implicated in angiogenesis in vitro using primary human ECs [13–15]. Moreover, a recent report suggested that *Kit* is expressed in embryonic hindbrain ECs during the period when it is vascularised by sprouting angiogenesis [4]. Therefore, we have systematically analysed whether KIT is expressed and has functional roles during organ vascularisation, which takes place in the mouse embryo from around E10.5 onwards. Our scRNA-seq analysis of brain tissue corroborated that *Kit* is expressed in ECs during the period of hindbrain angiogenesis (Fig. 2). However, KIT surface protein was detected in only a small subset of hindbrain ECs (Fig. 3), and hindbrain vascularisation was not obviously affected by KIT loss at E12.5 (Fig. 4).

It remains to be established whether *Kit* expression in a subset of brain ECs is related to haematopoiesis via endothelial-to-hematopoietic transition (endoHT) in the embryonic head. At E12.5, our scRNA-seq analysis failed to identify transcripts for the hemogenic EC marker *Runx1* in brain ECs (Fig. S2), and the more sensitive qRT-PCR assay identified only very low amounts of *Runx1* mRNA in brain ECs, which, together with neural cells, were the cell population with the lowest levels of *Runx1* (Fig. 2). On the one hand, low-level *Runx1* expression may support the idea that a rare population of embryonic brain ECs undergoes endoHT, as previously suggested [37–39]. On the other hand, low *Runx1* transcript levels may not necessarily reflect RUNX1 protein presence to imply biological function, because it was previously shown that genes with low-level transcript in a cell type are more likely to be non-functional [40]. Moreover, a prior report localised the presumed hemogenic progenitors of the embryonic head not to the neuroepithelium but to the branchial arch mesenchyme, which also contains the carotids, although this distinction was not explicitly commented on (see Fig. 4 in [39]). Taken together, there is currently insufficient evidence to localise the co-called hindbrain and branchial arch hematopoietic progenitors to the neuroepithelium. Nevertheless, *Kit* expression in neural progenitors agrees with prior lineage tracing in the *Kit*^*CreERT2*^; *Rosa*^*tdTom*^ hindbrain after tamoxifen induction at E8.5 [3]. Accordingly, neural progenitors express *Kit* in the mouse brain from around E8.5 onwards, and *Kit* expression is maintained to at least E12.5. It would, therefore, be interesting to examine whether such KIT expression contributes to neural progenitor expansion in the brain, in analogy to KIT’s role in hematopoietic progenitor expansion in the yolk sac and foetal liver [10], or whether KIT regulates neuronal behaviour, in analogy to its role the peripheral nervous system, where it promotes the survival and neurite outgrowth of sensory neurons in the dorsal root ganglia [41].

*Kit* is abundantly expressed in adult hepatic sinusoidal ECs (Fig. 1) [17], with a gradual increase in protein expression along the portal-central liver lobule axis [18]. Therefore, we examined whether foetal liver angiogenesis depends on KIT. However, only a small proportion of E12.5 foetal liver ECs expressed KIT surface protein, whereas many hematopoietic foetal liver cells were strongly positive for KIT at this stage (Fig. 3). Low-level KIT protein expression in E12.5 foetal liver ECs is consistent with the inability to detect *Kit* transcript in ECs by scRNA-seq, whereas *Kit* transcripts were readily detectable in foetal liver hematopoietic progenitors at this stage [10]. Accordingly, robust *Kit* expression in liver ECs, as observed in the adult (Fig. 1) [17], is acquired only later on during development, perhaps after the liver vasculature has specialised into the sinusoids that connect the portal triads to the central veins [42]. As KIT loss also did not affect the formation of liver vasculature at midgestation, it remains to be established whether KIT instead has a role in adult liver endothelium, for example after injury to promote regeneration from progenitor cell populations.

As lung ECs contained higher KIT levels than brain ECs at E12.5, E16.5, and 18.5 (Figs. 3, S5), we also examined lung vascularisation in E12.5 KIT-null embryos but found it to be unaffected (Fig. 4). Despite KIT being dispensable for lung embryonic angiogenesis, KIT was highly expressed in microvascular ECs of the adult lung (Fig. 1). Interestingly, recent studies suggested that KIT^+^ ECs in the adult lung microvasculature correspond to a population of specialised stem/progenitor cells that replenish the alveolar capillary endothelium during maintenance and repair, perhaps in response to KITL secreted from differentiated alveolar ECs themselves [19, 43]. Finally, we found that E12.5 limb vascularisation was unaffected by KIT loss. The absence of vascular defects in all *Kit*-null embryo organs examined at midgestation was unexpected, because KIT is expressed in embryonic ECs (Fig. 1–2) and marks a cell lineage that contributes to developing brain vasculature [3]. Moreover, decreased KIT expression reduces angiogenesis in mouse models of ocular pathology [15] and tumour growth [16]. The KIT requirement for angiogenesis under hypoxic and inflammatory pathological conditions has been ascribed to the hypoxia-induced upregulation of KIT protein levels, which activates β-catenin pro-angiogenic signalling in ECs [15], potentially indicating a selective role for KIT in postnatal or pathological angiogenesis, as opposed to embryonic angiogenesis.

Anaemia in KIT-null embryos precluded a definitive conclusion whether KIT expression in organ ECs was functionally relevant for vascular patterning late in gestation. Nevertheless, the opposing vascular phenotypes observed in the E18.5 brain versus lung and liver (Fig. 4), absent correlation of vascular phenotypes with the KIT^+^ EC proportion in these organs at that stage (Fig. S5), and the lack of vascular defects prior to erythrocyte deficiency at E12.5 (Fig. 4) together argue against a direct role for KIT in blood vessel growth during embryogenesis. Thus, in the absence of KIT, vascular density was increased in the E18.5 brain, where the proportion of KIT expression is low, but decreased in the E18.5 lung, where the proportion of KIT expression is high (Fig. 4). As the increased vascular density in the mutant brain was not explained by increased EC proliferation (Fig. S6), it may be a consequence of reduced vascular pruning. The finding that hypoxia-regulated genes are not upregulated in the E16.5 and E18.5 brain of KIT-deficient mice suggests that their brain remains adequately supplied by oxygen despite erythrocyte deficiency, possibly because of predominant glycolytic metabolism rather than oxidative phosphorylation across this organ, and in agreement with this organ being relatively spared from the growth defects caused by erythrocyte deficiency (Fig. S6). Nevertheless, reduced erythrocyte numbers in the circulation of late gestation embryos would be expected to affect vascular patterning in at least some organs. In agreement with this idea, E18.5 foetal liver vasculature is normally packed with erythrocytes and appears dilated compared to brain vasculature, whereas liver vasculature in E18.5 KIT-deficient embryos show significantly reduced vessel diameter despite a similar EC proliferation rate (Fig. 4), conceivably as a consequence of not being packed with nascent erythrocytes.

We conclude that KIT expression in ECs of developing organs does not reflect an obvious functional role in tissue vascularisation at midgestation, prior to embryonic anaemia, but that vascular anomalies appear in late-stage embryos concurrent with emerging anaemia. Therefore, the haemato-vascular requirement for KIT during development appears to be restricted to the hematopoietic system, whereby abnormal development indirectly impacts vascular development. Our findings do not exclude that KIT expression in adult endothelium is functionally important for organ repair and regeneration.

**Methods**: See Supplementary Information.

## Supplementary Information

Below is the link to the electronic supplementary material.

Supplementary file1 (PDF 844 kb)

## Data Availability

Publicly available datasets were analysed in this study: adult mouse scRNA-seq (https://tabula-muris.ds.czbiohub.org/, GSE109774); midbrain scRNA-seq (https://www.ncbi.nlm.nih.gov/geo/, GSE76381); embryonic lung scRNA-seq (https://www.ncbi.nlm.nih.gov/geo/, GSE165063, GSE160876); foetal liver scRNA-seq (https://ngdc.cncb.ac.cn/gsa/, CRA002445).
