# Supplementary material for: KIT is dispensable for physiological organ vascularisation in the embryo
Source: Angiogenesis. 2022 Apr 13;25(3):343–53. doi: 10.1007/s10456-022-09837-6 (PMC9249691; doi:10.1007/s10456-022-09837-6)
Supplement: Supplementary file 1 — Supplementary file1 (PDF 844 kb) [file 10456_2022_9837_MOESM1_ESM.pdf]

Supplemental files:

Supplemental figures and figure legends

Materials and Methods

Supplemental references

Supplemental figures and figure legends

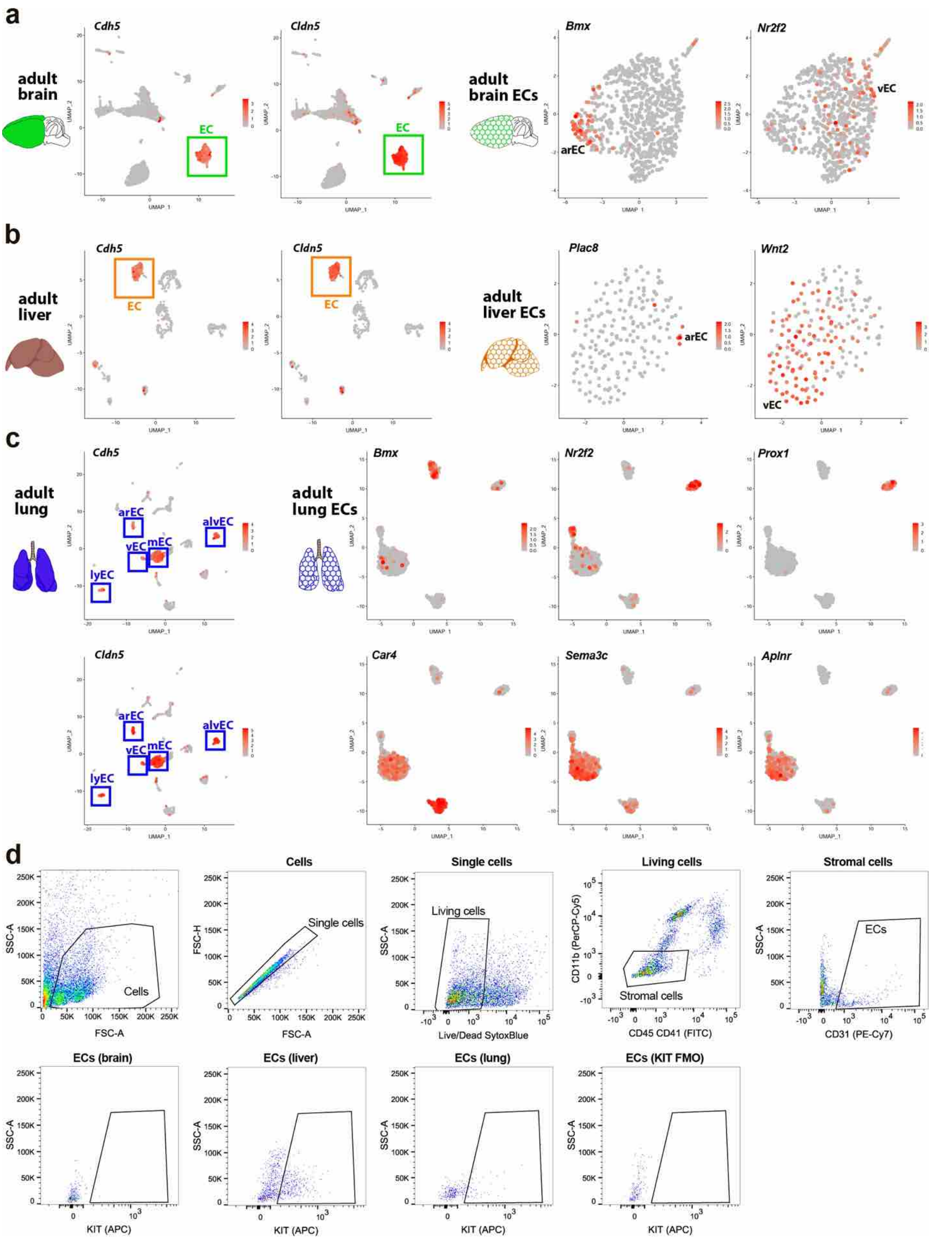

Figure S1. Characterisation of adult brain, liver and lung scRNA-seq datasets and KIT cell surface expression in adult brain, liver and lung ECs**.**

(**a-c**) scRNA-seq analysis of whole organ (left panels) and ECs (right panels) from adult mouse brain

(**a**), liver (**b**) and lung (**c**), including schematic representations of each organ. UMAP plots show *Cdh5* and *Cldn5* in whole organs, *Bmx* and *Nr2f2* in the brain EC population and *Plac8 and Wnt2* transcript levels in the liver EC population as representative markers for arterial (arEC) and venous ECs (vEC) [1], and *Bmx, Nr2f2, Prox1, Car4, Sema3c and Aplnr* transcript levels in the lung EC population as representative markers for arterial (arEC), venous ECs (vEC), lymphatic (lyEC), alveolar (alvEC) and microvascular (mEC) [2,1,3,4].

(**d**) Gating strategies for flow cytometry analysis of adult brain, liver and lung.

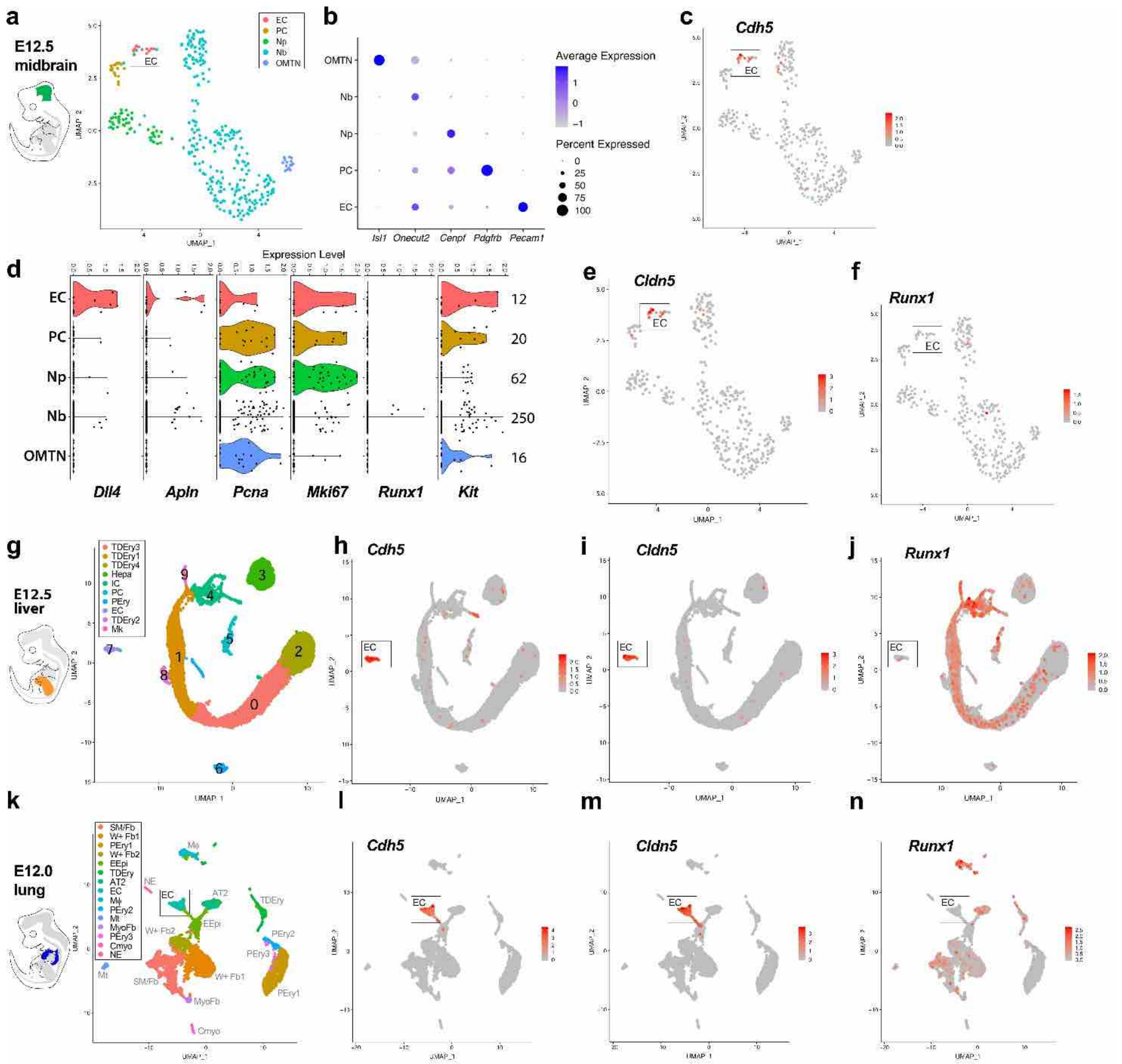

Figure S2. Endothelial cluster identification and *Runx1* expression in embryonic mouse midbrain, liver and lung.

scRNA-seq analysis of E12.5 mouse midbrain (**a-f**), liver (**g-j**) and E12.0 mouse lung (**k-n**), including schematic representations of each organ. UMAP plots visualise clusters of distinct cell types (**a,g,k**), as well as *Cdh5* (**c,h,l**), *Cldn5* (**e,i,m**) and *Runx1* (**f,j,n**) transcript levels in each cell cluster, whereby the colour intensity represents the average transcript level; boxes indicate EC clusters. Key marker genes for the indicated cell types are shown as bubble plots (**b**), whereby the dot size corresponds to the percentage of cells in which that marker was detected. Violin plots (**d**) illustrate transcript levels for the indicated genes, with the cell number in each cluster indicated to the right. Abbreviations: AT2, alveolar type 2; CMyo, cardiomyocyte; EEpi, early epithelium; MΦ, macrophages; Mg, microglia; Mt, mesothelium; MyoFb, myofibroblast; Nb, neuroblasts; NE, neuroendocrine; Np, neural progenitors; OMTN, oculomotor/trochlear nucleus; PC, pericytes; PEry1, primitive erythroid 1; PEry2, primitive erythroid 2; PEry3, primitive erythroid 3; SM/Fb, smooth muscle/fibroblasts; TDEry, transient definitive erythroid; W+ Fb1, WNT2+ fibroblasts 1; W+ Fb2, WNT2+ fibroblasts 2.

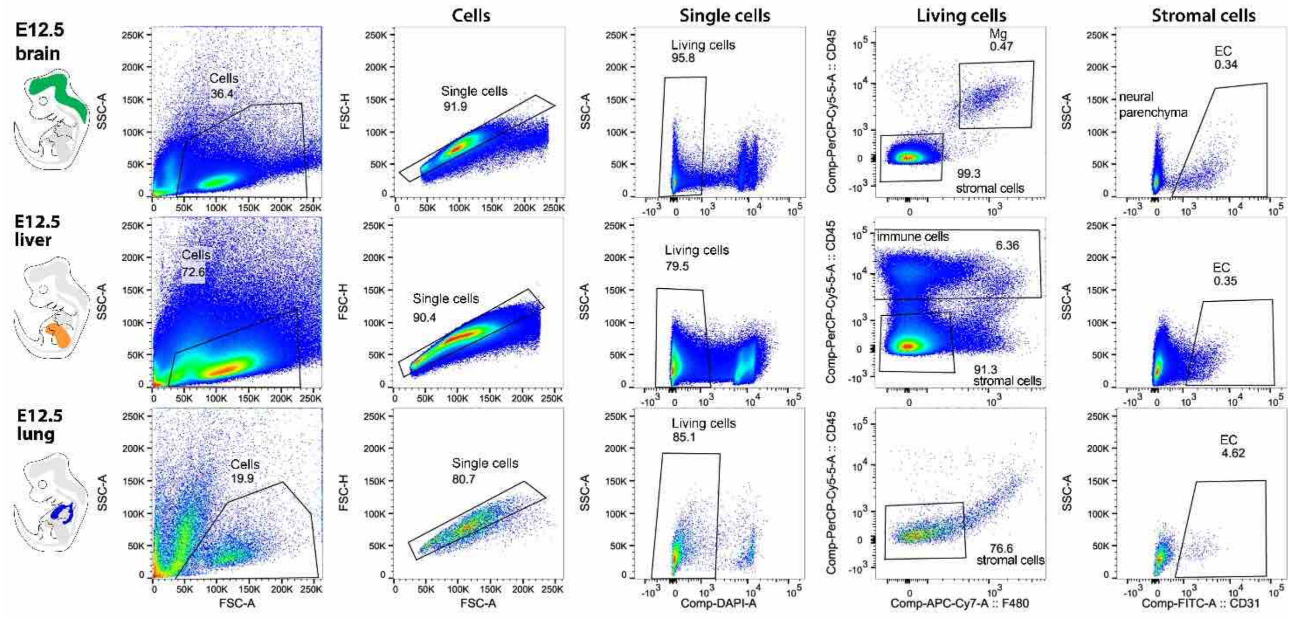

Figure S3. Gating strategy for E12.5 scRNA-Seq validation by qRT-PCR.

Gating strategy with the indicated markers to FACS-isolate the following cell populations: ECs (PECAM1^{+} CD45^{-} F4/80^{-}), microglia (F4/80^{+} CD45^{+}) and neural parenchyma (PECAM1^{-} CD45^{-} F4/80^{-}) from brains (including both midbrain and hindbrain, top); ECs (PECAM1^{+} CD45^{-} F4/80^{-}) and immune cells (CD45^{+}) from livers (middle); and ECs (PECAM1^{+} CD45^{-} F4/80^{-}) from lungs (bottom).

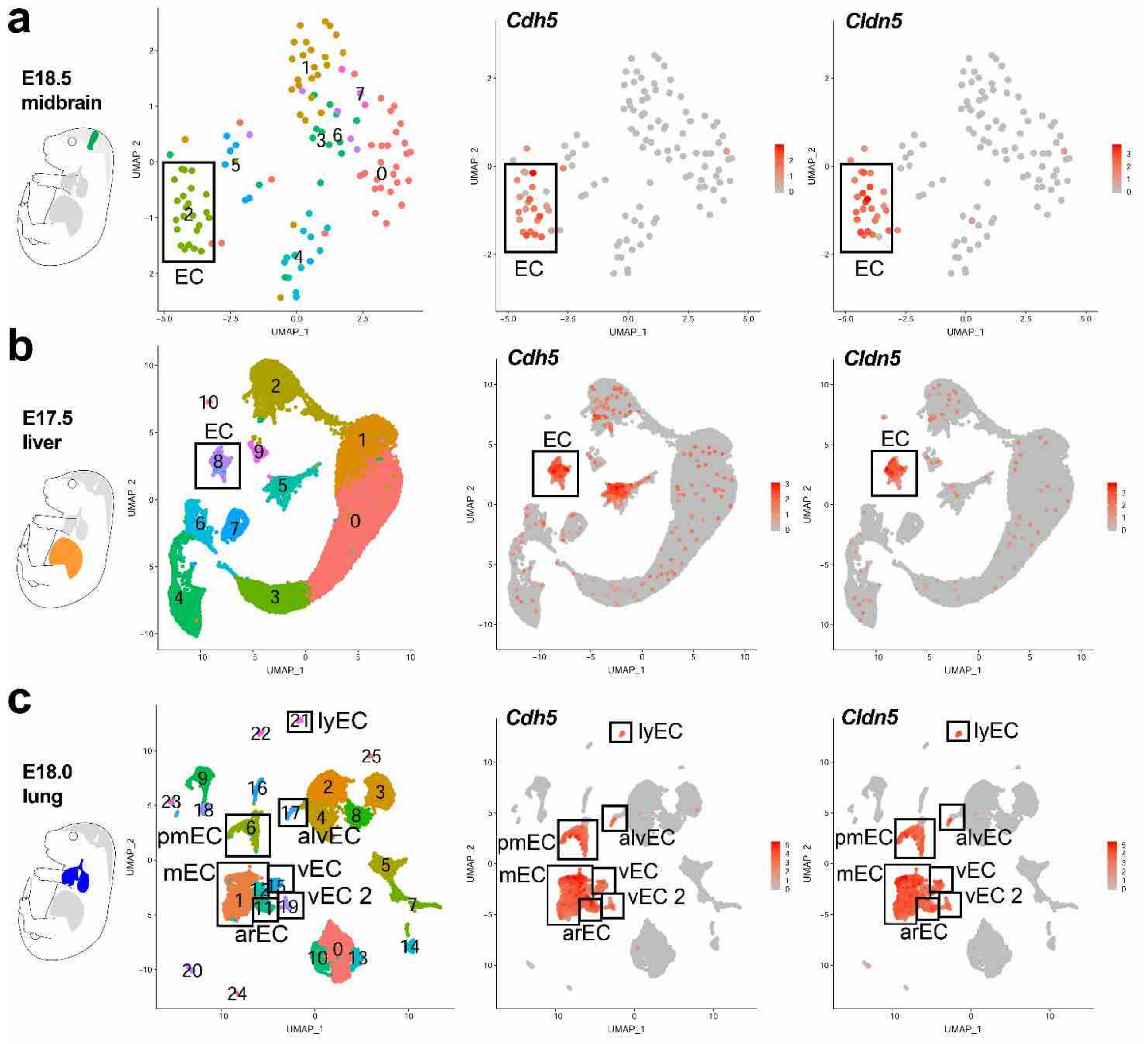

Figure S4. Endothelial cell cluster identification in late gestation mouse brain, liver and lung.

scRNA-seq analysis of E18.5 mouse midbrain (**a**), E17.5 mouse liver (**b**) and E18.0 mouse lung (**c**), including schematic representations of each organ. UMAP plots visualise clusters of distinct cell types (left panels) as well as *Cdh5* and *Cldn5* transcript levels in each cell cluster; boxes indicate the EC clusters. Abbreviations: alvEC, alveolar ECs; arEC, arterial ECs; lyEC, lymphatic ECs; mEC, microvascular ECs; pmEC, proliferating microvascular ECs; vEC, venous ECs ; vEC 2, venous ECs 2.

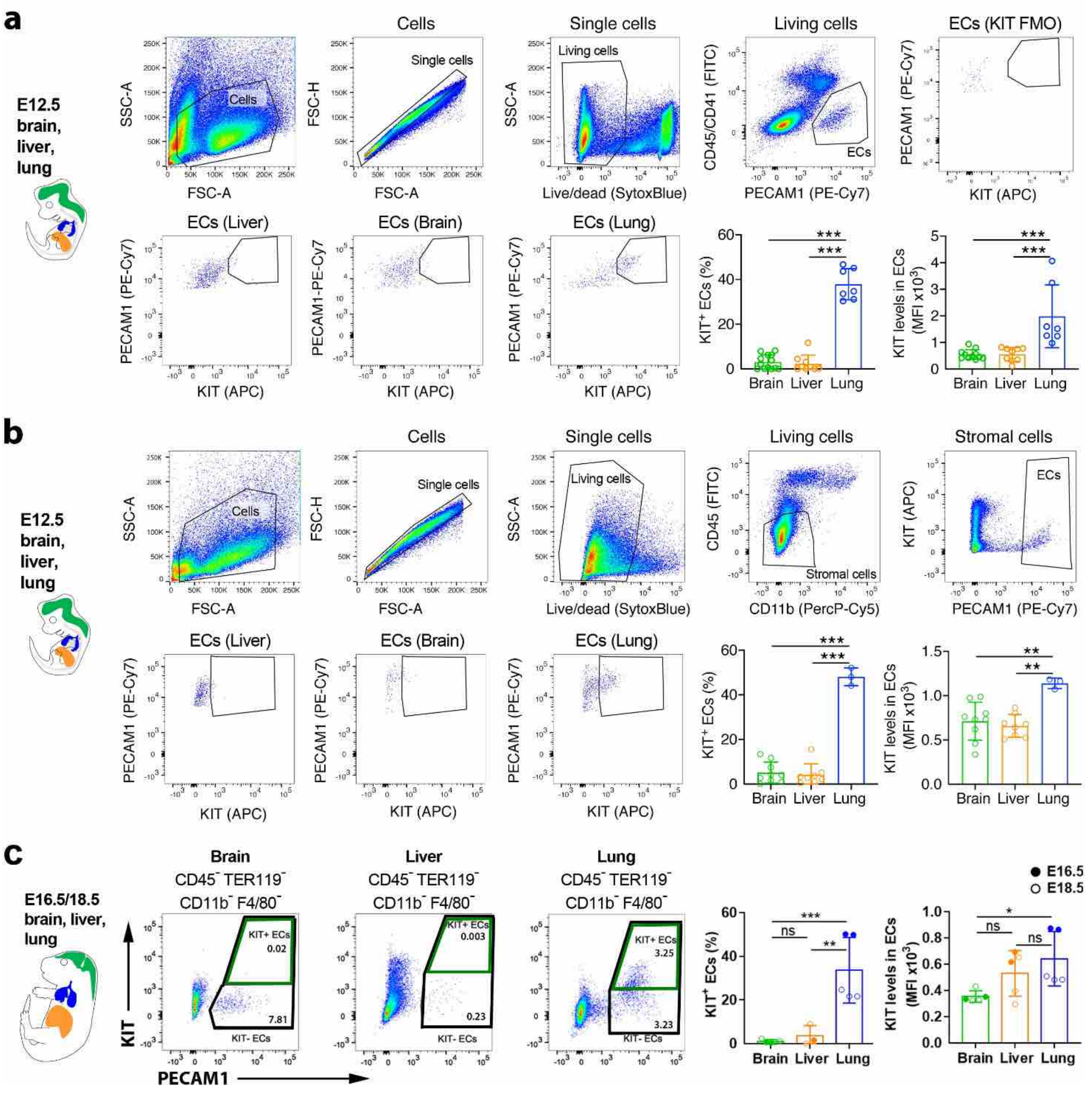

Figure S5. KIT cell surface expression in embryonic brain, liver and lung ECs**.**

(**a,b**) Gating strategies for flow cytometry analysis with CD41, CD45, PECAM1 and KIT (**a**) or CD11b, CD45, PECAM1 and KIT (**b**) of E12.5 mouse brain (pooled midbrain and hindbrain), liver and lung; brain n = 13 in (**a**), n = 9 in (**b**); liver, n = 9 in (**a**), n = 8 in (**b**); and lung, n = 7 in (**a**), n = 3 in (**b**). The bar graphs show the proportion of KIT$^+$ PECAM1$^+$ cells amongst all PECAM1$^+$ cells and the quantification of KIT protein surface levels as mean fluorescent intensity (MFI) in PECAM1$^+$ cells. All bar graph data show the mean ± SD; each data point represents the value from one embryo; **p<0.01, ***p<0.001 (one-way ANOVA followed by Tukey's multiple comparisons test).

(**c**) Flow cytometry analysis of live CD45$^-$ TER119$^-$ CD11b$^-$ F4/80$^-$ cells from E16.5 and 18.5 mouse brain, liver and lung to determine the proportion of KIT$^+$ ECs. Flow cytometry dot plots show the gating strategy for PECAM1 and KIT for each organ. Bar graphs show the proportion of KIT$^+$ PECAM1$^+$ cells amongst all PECAM1$^+$ cells and the quantification of KIT protein surface levels as mean fluorescent intensity (MFI) in PECAM1$^+$ cells (E16.5: n = 2; E18.5: n = 3). All bar graph data show the mean ± SD; each data point represents the value from one embryo; ns, p>0.05; **, p<0.01; ***, p<0.001 (two-way ANOVA followed by Tukey's multiple comparisons test).

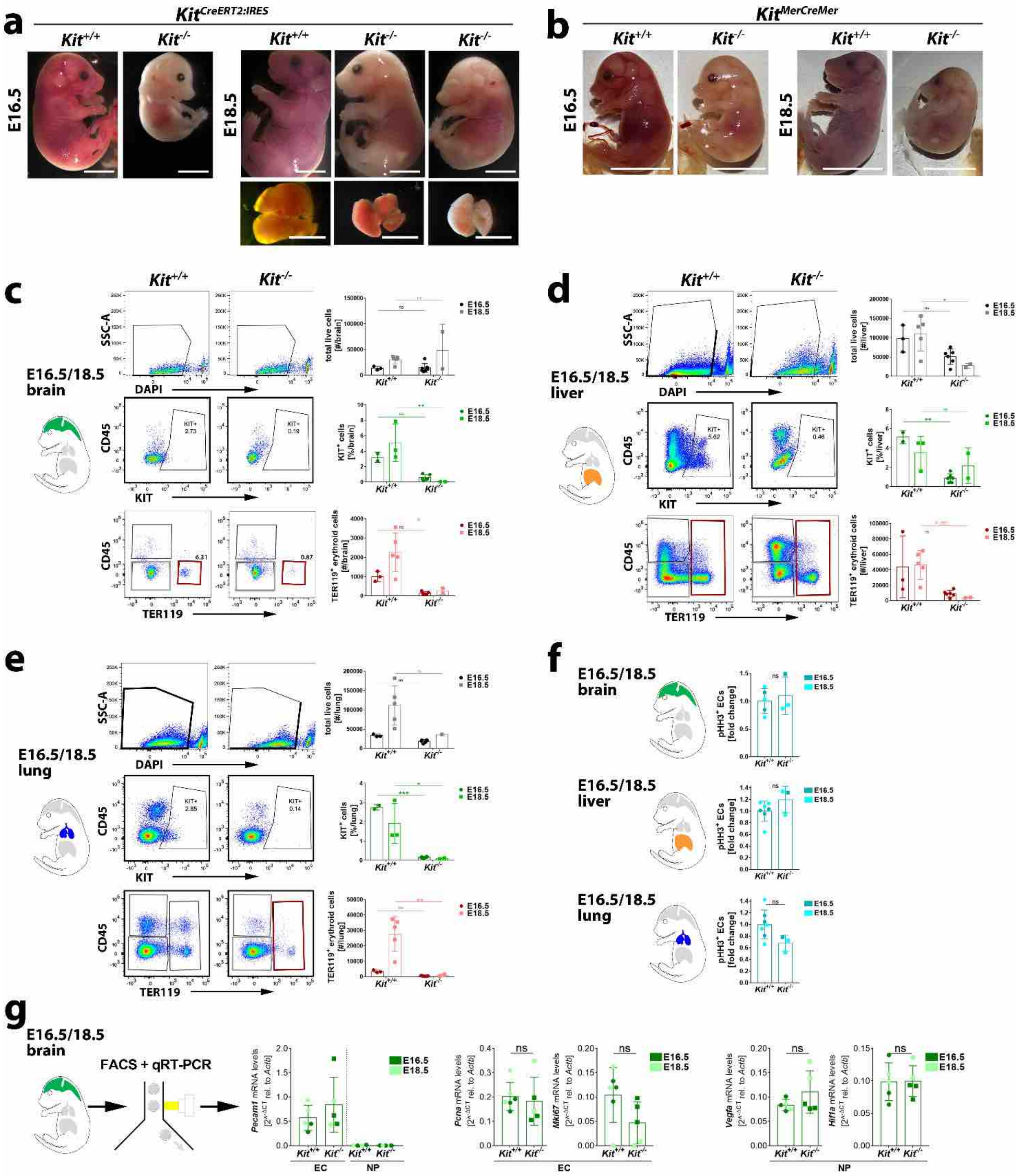

Figure S6. Phenotyping of late gestation *Kit*-null mutants.

(**a,b**) Micrographs of representative E16.5 and E18.5 mutants with the indicated *Kit* alleles, including littermate wild type controls; the corresponding liver micrographs are shown below each E18.5 embryo in (**a**). Scale bars: 0.5 cm (**a**); 1 cm (**b**).

(**c-e**) Flow cytometry analysis for the indicated markers from E16.5 ($Kit^{+/+}$ n = 3; $Kit^{-/-}$ n = 6) and E18.5 ($Kit^{+/+}$ n = 5; $Kit^{-/-}$ n = 2) mouse brain (**c**), liver (**d**) and lung (**e**), except for the quantification of KIT^{+} cells (E16.5 $Kit^{+/+}$ n = 2; E18.5 $Kit^{+/+}$ n = 3). Flow cytometry dot plots show the gating strategy for the

indicated parameters and markers. All bar graph data show the mean ± SD; each data point represents the value from one embryo; ns, p>0.05; *, p<0.05, **, p<0.01; ***, p<0.001 (two-way ANOVA followed by Tukey's multiple comparisons test).

(**f**) Quantification of phosphorylated histone H3 (pHH3)-positive ECs in E16.5 and E18.5 organs from wild type and *Kit*-null mutants from 1 litter for each time point; n = 5 wild type brains, n = 7 wild type livers and lungs and 3 KIT-null mutant brain, livers and lungs each.

(**g**) qRT-PCR analysis for *Pecam1*, the proliferation-related genes *Pcna* and *Mki67* and the hypoxia-regulated genes *Vegfa* and *Hif1a* in ECs and neural parenchyma (NP) cell populations isolated by FACS from mouse brains of E16.5 and E18.5 *Kit*-null and littermate wildtype controls (E16.5: $Kit^{+/+}$ n = 2, $Kit^{-/-}$ n = 3; E18.5: $Kit^{+/+}$ n = 3, $Kit^{-/-}$ n = 2). All bar graph data show the mean ± SD; each data point represents the value from one embryo.

Methods

Single cell RNA sequencing (scRNA-seq) analysis

Raw data for the adult brain, lung and liver scRNA-seq datasets from Tabula Muris, the E12.5 and E18.5 midbrain, the E12.0 and E18.0 lung the E13.0 and E17.5 liver, scRNA-seq datasets were obtained from GSE109774, GSE76381, GSE165063, GSE160876 (NCBI GEO) and CRA002445 (NGDC GSA), respectively [5,6]. Analyses were performed with RStudio v.1.3.1056. The raw gene expression matrices (UMI counts per gene per cell) were filtered, normalised and clustered using the R package Seurat v.3.2.0. Cells containing less than 200 feature counts and genes detected in less than 3 cells were removed, obtaining 1868 cells for the adult lung, 5302 for the adult brain, 919 for the adult liver, 6225 cells for E12.0 lung, 9033 cells for E18.0, 350 for the E12.5 midbrain, 121 cells for the E18.5 midbrain, 17806 for E13.0 liver and 27446 cells for E17.5 liver datasets. Downstream analysis included data normalisation ("LogNormalize" method and scale factor of 10,000) and variable gene detection ("vst" selection method, returning 2,000 features per dataset). The principal components analysis (PCA) was performed on variable genes, and the optimal number of principal components, PCs, for each sample was chosen using the elbow plot (40 for E18.5 midbrain, adult brain and liver, 50 for adult lung, 32 for E12.5 lung, 35 for E18.0 lung, 30 for E17.5 liver, 20 for E13.0 liver and 15 for E12.5 midbrain datasets). The selected PCs were used for Louvain graph-based clustering at a resolution of 0.3 for adult organs, 0.2 for E12.0 and 0.4 for E18.0 lung datasets, 0.1 for both E17.5 and E13.0 liver datasets, 1.5 for the E18.5 and 5.5 for the E12.5 midbrain datasets. Uniform manifold approximation and projection (UMAP) was chosen as a non-linear dimensionality reduction method, and cluster cell identity was assigned by manual annotation based on known marker genes.

Mouse strains

To obtain mouse embryos of defined gestational age, mice were paired in the evening and the presence of a vaginal plug the following morning was defined as E0.5. Embryos lacking *Kit* expression were generated by mating *Kit*$^{CreERT2:IRES}$ mice (MGI:5543260) [7] or by mating *Kit*$^{CreERT2:IRES}$ mice carrying *Rosa*^{tdTom} (MGI:3809524) [8] and *Csf1r-iCre* (MGI:4429470) [9] to obtain *Csf1r-iCre;Rosa*^{tdTom}*;Kit*$^{-/-}$ embryos. For some experiments at E16.5 and E18.5, we also used generated embryos lacking KIT by mating *Kit*^{MerCreMer} mice (MGI:5571475) [10]. All mouse strains were maintained on a mixed background (C57Bl6/J;129/Sv;FVB).

Flow cytometry

Tissues were mechanically and enzymatically homogenised in RPMI1640 with 2.5% fetal bovine serum (FBS, ThermoFisher), 1 mg/ml collagenase/dispase (Roche), 50 µg/ml DNase (Qiagen) and 100 µg/ml heparin (Sigma), incubated for 15 min with 0.5 mg/ml rat Fc block (Becton Dickinson) and labelled with a combination of FITC-conjugated rat anti-CD45 (1:200, clone 30-F11, cat 103108), FITC-conjugated rat anti-CD41 (1:100, clone MWReg30, cat 133903), APC-conjugated rat anti-KIT

(1:100, clone 2B8, cat 105812), PerCp-Cy5.5-conjugated rat anti-CD11b (1:100, clone M1/70, cat 101227), PE-Cy7-conjugated rat anti-PECAM1 (1:100, clone MEC13.3, cat 102524), APC-Cy7-conjugated rat anti-F4/80 (1:100, clone BM8, cat 123118), PerCP-Cy5-conjugated rat anti-CD45 (1:200, clone 30-F11, cat 103132), FITC-conjugated rat anti-PECAM1 (1:100, clone MEC13.3, cat 102506), PE-Cy7-conjugated rat anti-TER-119 (1:100, cat 116222) (all BioLegend) and PE-conjugated CD11b (1:200, clone M1/70, cat 50-0112, Tonbo Biosciences). Appropriate fluorescence gating parameters were established with unstained and fluorescence-minus-one (FMO) staining. In all experiments, doublets were eliminated using pulse geometry gates (FSC-H versus FSC-A and SSC-H versus SSC-A), whereas dead cells were removed using SYTOX Blue or 4′,6-diamidino-2-phenylindole (DAPI) (all Life Technologies). Single-cell suspensions were analysed using the BD LSRFortessa X-20 cell analyser, the BD Influx cell sorter or FACS Aria II (BD Biosciences); FlowJo v10.6.2 software (Tree Star/FlowJo LLC) was used for subsequent analyses.

FACS followed by quantitative RT-PCR (qRT-PCR) analysis

For FACS sorting, brains, livers and lungs from embryos were dissected, minced and digested in digestion mix as described above. Cells were incubated with Fc block (1:100, cat 101302, BioLegend) prior to staining with fluorescent antibodies for 20 min on ice. Cells were then incubated with APC-Cy7-conjugated rat anti-F4/80 (1:100, clone BM8, cat 123118, Biolegend), PerCP-Cy5-conjugated rat anti-CD45 (1:200, clone 30-F11, cat 103132, Biolegend), PE-Cy7-conjugated rat anti-TER-119 (1:100, cat 116222, Biolegend), PE-conjugated CD11b (1:200, clone M1/70, cat 50-0112, Tonbo Biosciences) and FITC-conjugated rat anti-PECAM1 (1:100, clone MEC13.3, cat 102506, Biolegend). After washing, cells were resuspended in FACS buffer and were incubated with DAPI before acquisition. Living single PECAM1$^+$ CD45$^-$ F4/80$^-$ ECs from all organs, triple negative neural parenchyma and CD45$^+$F4/80$^+$ microglial cells from brains and CD45$^+$ immune cells from livers were sorted for high purity on a FACS Aria II (BD Biosciences) directly into RNA lysis buffer (RLT buffer, QIAGEN), vortexed and immediately frozen at −80°C. For EC sorting from E16.5 and E18.5 brains, we additionally selected against CD11b and TER119. The RNeasy Micro kit (QIAGEN) was used to extract RNA and remove genomic DNA from sorted cells. RNA was reverse transcribed using the High-Capacity cDNA reverse Transcription Kit (Applied Biosystems, Thermofisher Scientific 4368814). Gene expression was measured by qRT-PCR using the Luna Universal qPCR Master Mix (M3003, NewEngland BioLabs) on a CFX Connect system (Bio-Rad). Gene expression was calculated relative to *Actb* according to the $2^{-\Delta CT}$ formula. Primer sequences:
Runx1-F 5’-CATCGCTTTCAAGGTGGTG-3’ and *Runx1*-R 5’-CATTTCTCAGTTCTGCCGAG-3; *Kit*-F 5’-GCCAGGAGAGACGCTGACTATC-3’ and *Kit*-R 5’-AGGCCTCGTATTCAACAACCA-3’; *Pecam1*-F 5’-CGATGCGATGGTGTATAACG-3’, *Pecam1*-R 5’-GTCACCTTGGGCTTGGATAC-3’; *Actb*-F 5’-AAGGCCAACCGTGAAAAGAT-3’ and *Actb*-R 5’-GTGGTACGACCAGAGGCATAC-3’; *Adgre1*-F 5’-TGGTTGAATACAGAGACGGG-3', *Adgre1*-R 5’-AAGGAGGACAGAGTTTATCGT-3'; *Vegfa*-F 5’-CAGATCATGCGGATCAAACCT-3', *Vegfa*-R 5’-TTGTTCTGTCTTTCTTTGGTCTG-3';

Hif1a-F 5'-AAACCACCCATGACGTGCTT-3' and *Hif1a*-R 5'-GAGCGGCCCAAAAGTTCTTC-3'; *Pcna*-F 5'-AGGAGGCGGTAACCATAGAGA-3' and *Pcna*-R 5'-AGACAGTGGAGTGGCTTTTGT-3'; *Mki67*-F 5'-ACCATCATTGACCGCTCCTT-3' and *Mki67*-R 5'-AGCTATCTCTTGAGGCTCGC-3'.

Wholemount tissue staining

Samples were fixed in freshly prepared or thawed 4% formaldehyde in PBS and processed as wholemounts for fluorescent staining as described previously for wholemount hindbrains [11]. Samples were blocked for 3 hours at room temperature with 10% donkey serum and 0.1% Triton-X in PBS prior to staining. For organs labelled with conjugated primary antibodies, we additionally added Fc block (1:200, cat 101302, BioLegend). We used the following antibodies and dilutions: rat anti-KIT (1:500; 553353, BD Pharmingen), rat anti-KIT APC-conjugated (1:200; clone 2B8, cat 105812, Biolegend), rat anti-PECAM1 FITC-conjugated (1:200; MEC13.3, Biolegend), rat anti-F4/80 (1:500; MCA497R, Serotec), rabbit anti pHH3 (1:1,000; 06-570, Millipore) and rabbit anti-RFP (1:1,000; PM005, MBL). Secondary antibodies used included donkey fluorophore-conjugated Fab fragments of anti-rabbit or rat IgG (Jackson ImmunoResearch). Biotinylated IB4 (1:200; L2140, Sigma) followed by Alexa-conjugated streptavidin (ThermoFisher) was used to detect brain ECs [12]. Images were acquired with a LSM710 (Zeiss) or an A1 (Nikon) laser scanning confocal microscope and processed using LSM image browser (Zeiss), Fiji (NIH Bethesda)[13] and Photoshop CS4 (Adobe Inc.) software. *Z*-stack projections of confocal images are shown unless indicated otherwise in the figure legends.

Statistical analysis

Tissues for analysis were allocated to experimental groups according to genotype, gestational age, organ or cell type, rather than being randomised. To ensure the unbiased interpretation of results, the genotype and gestational age were disclosed only after data collection was complete, but the investigators knew the sample origin (i.e., organ or cell type). No statistical methods were used to predetermine sample size. For hindbrain experiments, the F4/80$^+$, tdTomato$^+$ and IB4$^+$ volumes were determined from confocal *z*-stacks of four randomly chosen 0.25 mm^2 regions on the lateral side of each hindbrain. The *z*-stacks were surface rendered with Imaris (Bitplane) to obtain the F4/80$^+$, tdTom$^+$ and IB4$^+$ volumes, and the F4/80$^+$ volume was then subtracted from both the IB4$^+$ and tdTom$^+$ total volumes to obtain the IB4$^+$ EC and tdTom$^+$ EC volumes before calculating the ratio of tdTom$^+$ to IB4$^+$ EC volume. The same confocal *z*-stacks were analysed with Angiotool [14] as maximum intensity projections to determine the percentage of vascular area relative to total tissue area and the number of vascular intersections. All counts obtained from one hindbrain were averaged to yield the value for that hindbrain. We used Angiotool to quantify vascular complexity (branchpoints) and area in maximum intensity projections of the E12.5 and E18.5 liver and lung (whole left lobe), E12.5 forelimb (0.15 mm^2 rectangular area corresponding to the middle digit) and E18.5 brain striatum. A MATLAB (2021b, MathWorks) code was used to adjust confocal 2D maximum intensity projection images, segment, skeletonise and measure the vessel diameter at several cross-sections

perpendicular to the centre skeletonised lines. For all experiments, the error bars represent the standard deviation of the mean. Comparison of medians against means justified the use of a parametric test; to determine whether two datasets were significantly different, we therefore calculated P values with a two-tailed unpaired Student's t-test; $P < 0.05$ was considered significant. Statistical analyses were performed with Excel 12.2.6 (Microsoft Office) or Prism 7 (GraphPad Software).

Supplemental references

1. Kalucka J, de Rooij L, Goveia J, Rohlenova K, Dumas SJ, Meta E, Conchinha NV, Taverna F, Teuwen LA, Veys K, Garcia-Caballero M, Khan S, Geldhof V, Sokol L, Chen R, Treps L, Borri M, de Zeeuw P, Dubois C, Karakach TK, Falkenberg KD, Parys M, Yin X, Vinckier S, Du Y, Fenton RA, Schoonjans L, Dewerchin M, Eelen G, Thienpont B, Lin L, Bolund L, Li X, Luo Y, Carmeliet P (2020) Single-Cell Transcriptome Atlas of Murine Endothelial Cells. Cell 180 (4):764-779 e720. doi:10.1016/j.cell.2020.01.015
2. Niethamer TK, Stabler CT, Leach JP, Zepp JA, Morley MP, Babu A, Zhou S, Morrisey EE (2020) Defining the role of pulmonary endothelial cell heterogeneity in the response to acute lung injury. eLife 9. doi:10.7554/eLife.53072
3. Schupp JC, Adams TS, Cosme C, Jr., Raredon MSB, Yuan Y, Omote N, Poli S, Chioccioli M, Rose KA, Manning EP, Sauler M, DeIuliis G, Ahangari F, Neumark N, Habermann AC, Gutierrez AJ, Bui LT, Lafyatis R, Pierce RW, Meyer KB, Nawijn MC, Teichmann SA, Banovich NE, Kropski JA, Niklason LE, Pe'er D, Yan X, Homer RJ, Rosas IO, Kaminski N (2021) Integrated Single Cell Atlas of Endothelial Cells of the Human Lung. Circulation. doi:10.1161/CIRCULATIONAHA.120.052318
4. Gillich A, Zhang F, Farmer CG, Travaglini KJ, Tan SY, Gu M, Zhou B, Feinstein JA, Krasnow MA, Metzger RJ (2020) Capillary cell-type specialization in the alveolus. Nature 586 (7831):785-789. doi:10.1038/s41586-020-2822-7
5. La Manno G, Gyllborg D, Codeluppi S, Nishimura K, Salto C, Zeisel A, Borm LE, Stott SRW, Toledo EM, Villaescusa JC, Lonnerberg P, Ryge J, Barker RA, Arenas E, Linnarsson S (2016) Molecular Diversity of Midbrain Development in Mouse, Human, and Stem Cells. Cell 167 (2):566-580 e519. doi:10.1016/j.cell.2016.09.027
6. Tabula Muris C, Overall c, Logistical c, Organ c, processing, Library p, sequencing, Computational data a, Cell type a, Writing g, Supplemental text writing g, Principal i (2018) Single-cell transcriptomics of 20 mouse organs creates a Tabula Muris. Nature 562 (7727):367-372. doi:10.1038/s41586-018-0590-4
7. Klein S, Seidler B, Kettenberger A, Sibaev A, Rohn M, Feil R, Allescher HD, Vanderwinden JM, Hofmann F, Schemann M, Rad R, Storr MA, Schmid RM, Schneider G, Saur D (2013) Interstitial

cells of Cajal integrate excitatory and inhibitory neurotransmission with intestinal slow-wave activity. Nature communications 4:1630. doi:10.1038/ncomms2626

8. Madisen L, Zwingman TA, Sunkin SM, Oh SW, Zariwala HA, Gu H, Ng LL, Palmiter RD, Hawrylycz MJ, Jones AR, Lein ES, Zeng H (2010) A robust and high-throughput Cre reporting and characterization system for the whole mouse brain. Nature neuroscience 13 (1):133-140. doi:10.1038/nn.2467
9. Deng L, Zhou JF, Sellers RS, Li JF, Nguyen AV, Wang Y, Orlofsky A, Liu Q, Hume DA, Pollard JW, Augenlicht L, Lin EY (2010) A novel mouse model of inflammatory bowel disease links mammalian target of rapamycin-dependent hyperproliferation of colonic epithelium to inflammation-associated tumorigenesis. The American journal of pathology 176 (2):952-967. doi:10.2353/ajpath.2010.090622
10. van Berlo JH, Kanisicak O, Maillet M, Vagnozzi RJ, Karch J, Lin SC, Middleton RC, Marban E, Molkentin JD (2014) c-kit+ cells minimally contribute cardiomyocytes to the heart. Nature 509 (7500):337-341. doi:10.1038/nature13309
11. Fantin A, Vieira JM, Plein A, Maden CH, Ruhrberg C (2013) The embryonic mouse hindbrain as a qualitative and quantitative model for studying the molecular and cellular mechanisms of angiogenesis. Nature protocols 8 (2):418-429. doi:10.1038/nprot.2013.015
12. Fantin A, Vieira JM, Plein A, Denti L, Fruttiger M, Pollard JW, Ruhrberg C (2013) NRP1 acts cell autonomously in endothelium to promote tip cell function during sprouting angiogenesis. Blood 121 (12):2352-2362. doi:10.1182/blood-2012-05-424713
13. Schindelin J, Arganda-Carreras I, Frise E, Kaynig V, Longair M, Pietzsch T, Preibisch S, Rueden C, Saalfeld S, Schmid B, Tinevez JY, White DJ, Hartenstein V, Eliceiri K, Tomancak P, Cardona A (2012) Fiji: an open-source platform for biological-image analysis. Nature methods 9 (7):676-682. doi:10.1038/nmeth.2019
14. Zudaire E, Gambardella L, Kurcz C, Vermeren S (2011) A computational tool for quantitative analysis of vascular networks. PloS one 6 (11):e27385. doi:10.1371/journal.pone.0027385
